# Home Visit Based Mindfulness Intervention for Vietnamese American Dementia Family Caregivers: A Pilot Feasibility Study

**DOI:** 10.31372/20200504.1096

**Published:** 2021

**Authors:** Christine Trinh Le, Jung-Ah Lee

**Affiliations:** a Tulane University, New Orleans, Louisiana, United States; b University of California-Irvine, Irvine, California, United States

**Keywords:** dementia, emotion, family caregivers, mindfulness, Vietnamese Americans

## Abstract

**Background:** Healthcare disparities continue to exist among the Vietnamese American (VA) community and many factors (e.g., fear of social stigma) deter family caregivers of persons with dementia (PWD) from seeking assistance.

**Purpose:** To pilot-test a language-specific and culturally appropriate mindfulness intervention to improve dementia VA family caregiver well-being.

**Methods:** Bilingual, trained research assistants administered a mindfulness exercise (i.e., deep breathing) to family caregivers and provided continuous support and care resources through weekly home visits for a month. Weekly surveys measured changes in emotion, feelings of connectedness to the PWD, and mood (i.e., happiness) before and after the intervention.

**Results:** A total of nine VA family caregivers of PWD participated in this pilot study. Positive affect showed an increasing trend (*M*_pre_ = 16.0 (SD = 3.48), *M*_post_ = 17.1 (SD = 3.06)) and negative affect showed a decreasing trend (*M*_pre_ = 6.44 (SD = 3.31), *M*_post_ = 5.22 (SD = 0.359)). Happiness showed an increasing trend (*M*_pre_ = 4.30 (SD = 0.767), *M*_post_ = 4.44 (SD = 0.873)).

**Conclusions:** These findings suggest that a home-based dementia family caregiver intervention with mindfulness exercises may potentially increase positive affect and decrease negative affect in Vietnamese American family caregivers of PWD. Similar interventions may help reduce caregiver burden in dementia family caregivers of other cultures.

## Introduction

As of 2017, an estimated 1.3 million Vietnamese Americans reside in the United States, with more than 65,000 over the age of 65 (Alperin & Batalova, 2018; Tran & Hinton, 2010). Healthcare disparities continue to exist among the Vietnamese community and are thought to be due in part to differences in culture, fear of social stigma, English-language barriers, distrust of the healthcare system, and unawareness of available resources (Augsberger, Yeung, Dougher, & Hahm, 2015; Xiao, Habel, & De Bellis, 2015).

Compared to other Asian American groups with different migration experiences to the United States, Vietnamese Americans were found to have overall poorer health (Frisbie, Cho, & Hummer, 2001). This may be due in part to the difficulties in adjusting from an economically disadvantaged country to a wealthy, highly industrialized country such as the United States (Cho & Hummer, 2010). Additional factors such as traumatic exposure to war-related violence and lower educational achievement are thought to contribute to adverse health outcomes and appear to increase the risk of cognitive impairment and depression (Kaup et al., 2014; Tschanz, Norton, Zandi, & Lyketsos, 2013). Additionally, with age, memory complaints become increasingly common, and the risk of dementia also increases (Meyers et al., 2018). Several studies have suggested that knowledge about dementia in Asian Americans is lacking, often with many misconceptions to which symptoms constitute dementia (Ho & Woo, 2013; Nguyen et al., 2016; Woo, 2013). Social and cultural factors may have an impact on how dementia is perceived, how the caregiver provides care, and whether caregivers seek help and support (Xiao et al., 2015). Lack of access to supportive services is correlated with increased caregiver burden, increased morbidity, increased mortality, and an increased risk of hospitalization of caregivers (Brodaty & Donkin, 2009; Low, Anstey, Lackersteen, & Camit, 2011). A better understanding of the barriers to healthcare in this population is an important step in developing a culture-appropriate approach to improve family caregiver burden.

Mindfulness-based stress reduction (MBSR) is a technique that aims to reduce stress and better manage emotions by focusing one’s attention on the present (Bishop, 2002; Chambers, Gullone, & Allen, 2009). A study suggested that mindfulness-based interventions improved overall health outcomes and reduced stress in family caregivers of persons with dementia (PWD) (Whitebird et al., 2013).

Mindfulness is ingrained in the teachings of Buddhism, which has deep roots in Vietnam, and it was thought to have been practiced since the first century c.e. (H?nh & Hanh, 2001; Le & Trieu, 2016). Buddhist teachings were widely accepted by the indigenous Vietnamese people and the practice of mindfulness has played a critical role in establishing modern Vietnamese culture (Le & Trieu, 2016). To our understanding, there is currently no study that has investigated the efficacy of an in-home mindfulness intervention in the Vietnamese population. Due to the extensive integration of mindfulness into Vietnamese culture, we believe that a mindfulness-based intervention would be effective and well-received by Vietnamese caregivers.

Classic MBSR is an intensive training program that involves eight weekly classes, an all-day silent retreat, daily and weekly home assignments, and over thirty hours of in-class contact (Santorelli, 2014). Due to the extensive time-commitment and travel requirements of the classical MBSR training, we developed a modified mindfulness procedure that involved 4 weeks of in-home visits instead of the traditional 8 weeks of training. Additionally, the modified mindfulness-based intervention addressed several problems unique to our target population (Vietnamese immigrants). Participants were the primary caregiver for PWD and could not be separated from the PWD for extended periods of time, participants reported relatively low English proficiency of 1.67 (where 0 = cannot speak English and 4 = excellent proficiency), and participants reported greater acculturation to Vietnam than to the United States. Therefore, we believe that a modified, in-home Vietnamese mindfulness-based intervention would be more effective with this population than the standard mindfulness-based intervention.

Data were collected with revised Positive and Negative Affect Schedule (PANAS).

Based on a previous study (Whitebird et al., 2013), we predict that our home-based mindfulness intervention (i.e., deep breathing exercise) will improve the emotional well-being of Vietnamese American family caregivers by reducing negative affect, increasing positive affect, and increasing the happiness and emotional intimacy between the caregivers and the PWD.

The purpose of this pilot feasibility study is to determine whether an in-home, language-specific mindfulness-based intervention will effectively improve positive affect and reduce negative affect in Vietnamese American family caregivers of PWD.

## Methods

This study was a pilot feasibility study that was a part of a larger parent study. The protocol of the parent study was approved by the Institutional Review Board on Human Subject Research at University of California-Irvine.

### Research Team Training

Student interventionalists were trained by the lead researcher with expertise in geriatrics and Alzheimer’s research (J.L.) with a variety of resources, including the Alzheimer’s and dementia training on-line courses administered by Alzheimer’s Association (Alzheimer’s Association), the in-person “Dementia Care Training for Professionals” course by Alzheimer’s Orange County (Alzheimer’s Orange County, 2019), and University of California, Los Angeles Caregiver training videos (UCLA Health). All training interventionalists were accompanied and supervised by senior interventionists for several home visits before being allowed to conduct home visits independently. The mindfulness (i.e, deep breathing) script was developed in English, and then a bilingual research assistant who completed university education in Vietnam translated the script into Vietnamese. Another bilingual research assistant double checked the quality of the translation and checked the script for ease of comprehension.

### Participants

Inclusion criteria included (a) were eighteen years or older, (b) were the primary caregiver of a family member diagnosed with dementia, (c) immigrated from Vietnam, (d) did not plan to put the PWD in long-term care within 6 months, (e) used a smartphone, (f) were able to receive and reply to text messages, and (g) were willing to wear a smartwatch to measure physiological data. Physiological data (i.e., heart rate variability) collected by the smartphone were presented in another paper (Lee, Labbaf, Rahmani, Kehoe, Dutt, 2019). Exclusion criteria included (a) cognitive impairment (as screened by MiniCog), (b) were undergoing active cancer treatment, or (c) had a diagnosis of an irreversible health condition that was likely to impact 6-month survival or the ability to participate in the study. The majority of the participants were monolingual (Vietnamese) with an average English proficiency of 1.67, where 0 = no English speaking ability and 4 = excellent proficiency.

### Recruitment

Recruitment occurred primarily through distribution of flyers by Vietnamese physicians and presentations at various Vietnamese caregiver support groups in Southern California. Recruitment was also conducted by the snowball recruitment method via word-of-mouth. Inclusion criteria and qualification for the study were confirmed by a follow-up phone conversation. Participants were compensated with $100 cash and a smartwatch for their time and participation.

### Intervention

A trained Vietnamese-speaking university student administered four weekly home visits individually with each participant. The study information sheet, which included the purpose and the procedures of the study, was provided to each participant. The focus of the intervention was the mindfulness-based training (i.e., deep breathing exercise), in which participants were taught and encouraged to use a deep breathing technique throughout the duration of the study. Participants were instructed to sit back and relax, inhale through the nose, count from one to six, and exhale through the mouth. The exercise was repeated for one minute each visit and participants were encouraged to practice deep breathing during times of stress and/or exhaustion. In addition, educational information was provided to the participant in Vietnamese according to each participant’s individual needs and concerns. Information included the progression of dementia, nutrition, stress-management for both the PWD and the caregiver, communication techniques, legal information, end-of-life care, and medical management. Participants were also provided information about local supportive services and programs, including community support groups. With feedback from each participant at the end of each session, subsequent visits were tailored more specifically to each participant.

### Data Collection

The participant’s consent was obtained at the initial visit. Participants completed a brief survey before and after the mindfulness exercise. Once weekly text message reminders to check-in and connect with the participant were sent to each participant throughout the entire period of the study, however, these responses were not collected as data.

### Measures

Emotions, happiness, and feelings of connectedness with the PWD were measured by PANAS, modified by two researchers who specialized in Alzheimer’s and aging research. To measure the effectiveness of the intervention, surveys were administered prior to the intervention and following the intervention at each of the four visits. The PANAS survey measured positive affect (PA) and negative affect (NA), as well as closeness to the PWD with a visual scale (where 1 = not close at all, 7 = very close) and current mood with a visual smiley face scale (where 1 = not happy at all, 5 = very happy) (Mackinnon et al., 1999; Watson & Clark, 1999; Watson, Clark, & Tellegen, 1988).

### Statistical Analysis

Various statistical methods were used to analyze the data. Measures of central tendency (mean (*M*), median, range) and variability (standard deviation (SD)) were calculated on the demographical data and on the data from the modified PANAS. Due to the small scale of the pilot study (*n* = 9), *t*-tests were performed on the data with a *p*-value of 0.1 or below indicating statistical significance. The sample size was determined on the basis of practical feasibility and the aim was to test the acceptability of a language-specific, culture-appropriate home-visit-based mindfulness intervention on immigrant Vietnamese family caregivers of PWD. Use of a smaller sample size was used to ensure that the aim could be completed in a timely manner. Therefore, a *p*-valve of 0.1 was used to indicate statistical significance because the subsequent larger, more comprehensive study would provide a control of the initial elevated error rate of the small-scale pilot study (Moore, Carter, Nietert, & Stewart, 2011; Schoenfeld, 1980).

## Results

Surveys were administered at the first home visit to collect demographic information and baseline data (i.e., positive affect, negative affect, feelings of connectedness with PWD, and happiness) from nine Vietnamese American dementia family caregiver participants about themselves and about the PWD. A summary of the characteristics of the participants and of the PWD are presented in Table 1 and Table 2 respectively.

**Table 1:** Characteristics of Family Caregiver Participants (n = 9)

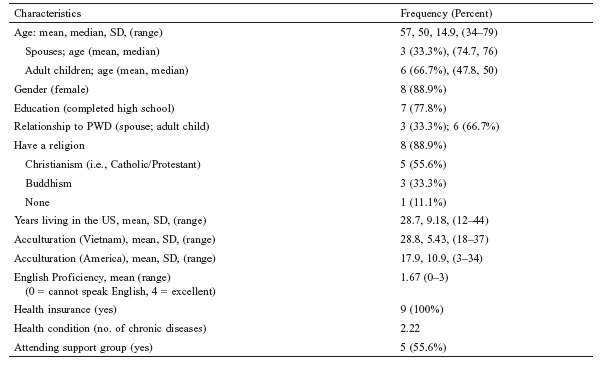

**Table 2:** Characteristics of Persons with Dementia (n = 9)

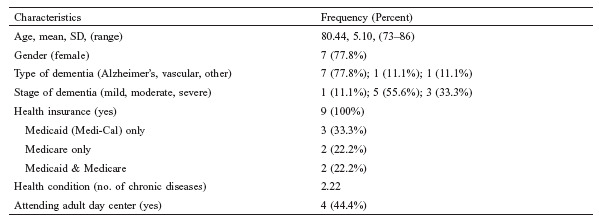

At every home visit, self-reported responses were recorded via surveys to measure caregiver affect, feelings of connectedness, and mood (happiness). In Table 3, summaries (mean, standard deviation) of data collected each week are presented. If baseline data was not available, data from the second visit were used in lieu of baseline data in the analysis.

**Table 3:** Summaries of Data per Home Visit

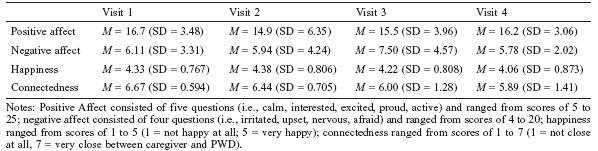

To test for statistical differences between baseline and final data, *t*-tests were conducted. A summary of preintervention and postintervention means (*M*), standard deviations (SD), and *t*-values are presented in Table 4.

**Table 4:** Differences in Positive Affect and Negative Affect in Pre-Post Interventions

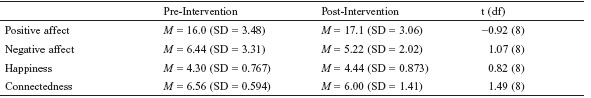

**Figure 1:**
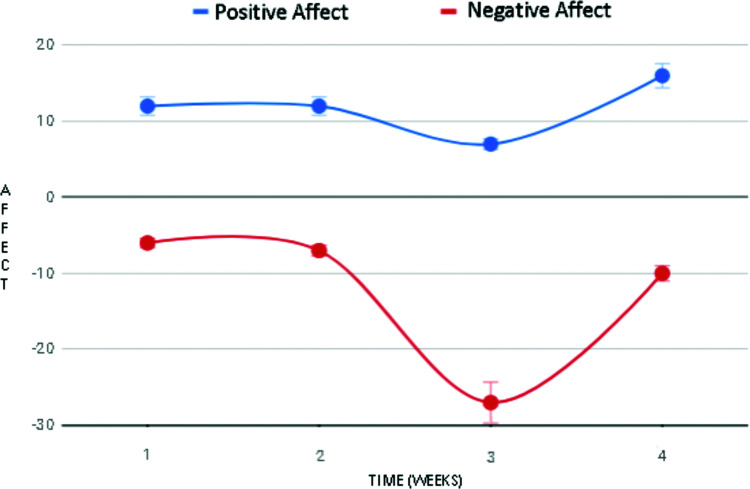
Changes in positive and negative affect.

**Figure 2:**
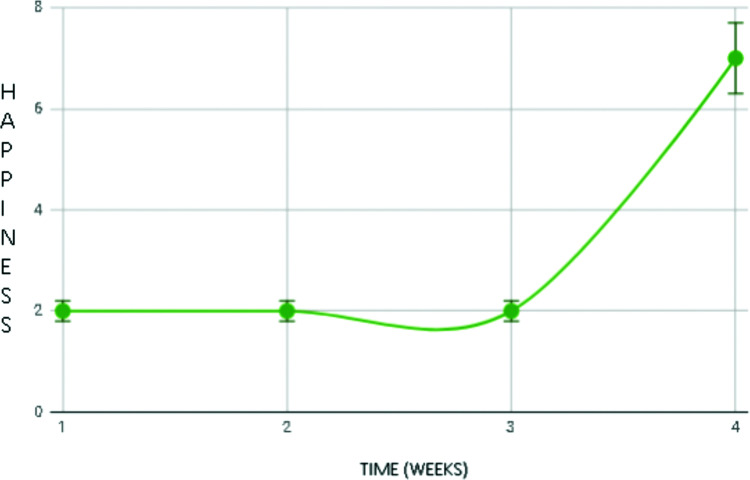
Changes in mood (happiness).

**Figure 3:**
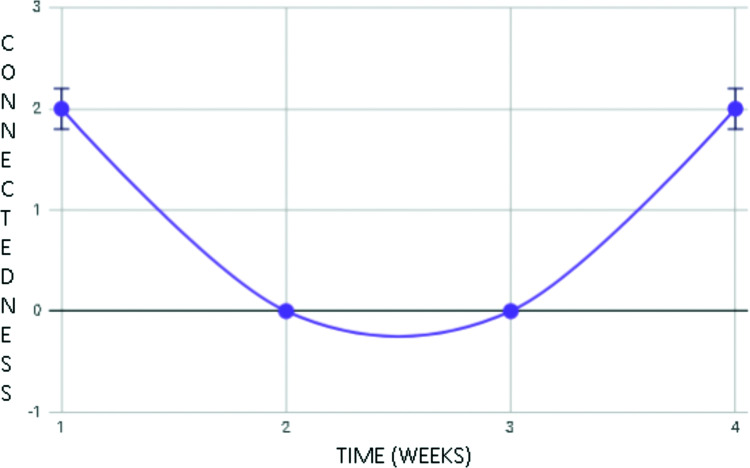
Changes in feelings of connectedness with PWD.

Positive affect (*M*_pre_ = 16.0 (SD = 3.48), *M*_post_ = 17.1 (SD = 3.06)), and happiness (*M*_pre_ = 4.30 (SD = 0.767), *M*_post_ = 4.44 (SD = 0.873)) showed increasing trends and negative affect showed a decreasing trend (*M*_pre_ = 6.44 (SD = 3.31), *M*_post_ = 5.22 (SD = 2.02)), Feelings of connectedness showed no change (*M*_pre_ = 6.56 (SD = 0.594), *M*_post_ = 6.00 (SD = 1.41)). Trends in affect, happiness, and connectedness are shown in Figures 1–3, respectively.

## Discussion

Many barriers (e.g., social stigma, cultural pressures, English-language barriers, and feelings of isolation) deter Vietnamese American family caregivers from seeking support; lack of support is associated with increased caregiver burden and morbidity (Augsberger et al., 2015; Brodaty & Donkin, 2009; Ho & Woo, 2013; Low et al., 2011; Nguyen et al., 2016; Woo, 2013; Xiao et al., 2015). With a growing number of Vietnamese Americans residing in the United States (Trueman, 2015), it is important to investigate the best methods to effectively provide support and care resources to better promote the well-being of PWD and their families.

A modified mindfulness-based intervention was administered in the participants’ home with self-reported data collected via a modified PANAS. The intervention was conducted in Vietnamese because the majority of the participants were monolingual (Vietnamese) with an average English proficiency of 1.67, where 0 = no English speaking ability and 4 = excellent proficiency. While the effects of a mindfulness-based intervention showed promising results in reducing stress in Vietnamese youth (Le & Trieu, 2016), to our understanding, there is currently no study that has investigated the effects of MBSR on Vietnamese family caregivers of PWD. The at-home, culturally sensitive mindfulness intervention showed promising results, with general increases in positive affect and happiness, as well as a general decrease in negative affect. An increasing trend in positive affect and happiness suggested that participants experienced improved positive emotion (e.g., calm, pride) and better overall mood. A decreasing trend in negative affect suggested that participants experienced fewer negative emotions (e.g., anger, anxiety). These findings are consistent with those of a previous study that administered interventions involving similar mindfulness-based techniques to family caregivers of PWD (Whitebird et al., 2013).

Baseline data (i.e., measured during the first week prior to the mindfulness intervention) was compared to postintervention data (i.e., measured following the mindfulness intervention during the second to fourth visits). An increasing trend (*y* = 5.9*x* + 26.9, *R*^2^ = 0.248) in positive affect, a decreasing trend (*y* = −9.9*x* + 0.448, *R*^2^ = 0.448) in negative affect, and an increasing trend (*y* = 1.5*x* + 1, *R*^2^ = 0.6) in happiness were observed. There was no change in feelings of connectedness toward the PWD. These results support earlier findings that in-person interventions correlated with a general decrease in caregiver burden and an increase in perceived health of caregivers of PWD (Martin-Carrasco et al., 2009; Whitebird et al., 2013).

Positive affect showed a slight increase from week 1 to week 2, then dipped below baseline during week 3, and improved during week 4. A similar trend was observed for happiness. The decrease between weeks 3 and 4 was unexpected, however, the small sample size (*n?*=*?*9) may have contributed to this result. Additionally, because the data was self-reported via surveys, uncontrolled factors may have influenced the results. For instance, one participant reported the hospitalization of a family member, which caused additional stress and concern, which may have affected survey results for that week. Negative affect showed an unexpected increase in between weeks 3 and 4, although final negative affect was lower than initial negative affect. As with the unexpected trend seen in positive affect, the small sample size as well as factors that were not controlled for could have contributed to this trend. Feelings of connectedness with the PWD showed an initial decreasing trend until approximately half-way through the intervention, and then showed an increasing trend. Because the participants were introduced to a large amount of information at once, participants may have felt initially overwhelmed and disconnected from the PWD. The increasing trend of connectedness in the later portion of the study is encouraging, but more research is needed to examine whether or not mindfulness interventions improve feelings of connectedness with PWD.

There were several limitations to the study. This pilot feasibility study was one part of a larger, more comprehensive parent study. In this study, only the effect of mindfulness on well-being was analyzed. Other factors, including education, may have contributed to the effects observed on participant well-being. This study involved a single-arm intervention without a control group. Therefore, the effects of other potential variables (e.g., level of education, age) on affect, mood, and feelings of connectedness could not be discerned from the effects of the intervention. Additionally, there may have been selection bias because all participants were volunteers. Study recruitment was conducted via distribution of flyers at physician offices, advertisements at support groups, and by the snowball effect (word-of-mouth). Thus, the study participants could be persons who are more motivated to seek out caregiving information; these persons may differ from persons who do not have motivation to seek additional care resources to improve care for the PWD. This study was conducted in a regional area where many Vietnamese Americans live and thus Vietnamese Americans who live elsewhere in the United States may not have the same opportunity to participate in a language- and culture-specific caregiver research. For these reasons, the sample of participants from this study may not be representative of the population as a whole. Therefore, the findings from this study should be considered with some limited generalizability. Due to these limitations, we recognize that our results may only apply to certain groups under certain conditions and may or may not generalize to the population.

Several changes can be made to improve the study design in the future. A larger sample size would help minimize the effect of selection bias on the results. Additionally, to minimize the effect of potential confounding variables on the results, the study participants should be randomized into groups. Having a control group (i.e., a group that is not instructed on the mindfulness/deep breathing technique) to compare the experimental group to would determine if the effects on affect, happiness, and connectedness were due to the intervention. A longitudinal study design could allow for observations on outcomes (e.g., mood, affect) to be made over a longer period of time. This allows for detection of changes between variables over time. In addition, later data can be compared to baseline data and can be more suggestive of a cause–effect relationship. While the time and cost of implementing such a design could be a deterrent, if future research supports that similar interventions lead to a reduction of caregiver burden (i.e., increased positive affect and happiness, decreased negative affect), then investments in such interventions to prevent or to lessen the effects of chronic conditions (e.g., depression) may actually be more cost effective than treating such conditions in high-risk populations such as in Vietnamese Americans (Kaup et al., 2014; Tschanz et al., 2013).

## Conclusion

A 4-week, home-based, culturally sensitive, language-specific intervention was conducted on nine Vietnamese American family caregivers of PWD with the intention of decreasing caregiver burden by increasing positive affect, happiness, and feelings of connectedness with PWD and decreasing negative affect. Positive affect and happiness showed an increasing trend, negative affect showed a decreasing trend, and feelings of connectedness did not change. The encouraging trends in positive affect, negative affect, and happiness in Vietnamese American dementia family caregivers suggest that similar mindfulness interventions have the potential to reduce caregiver burden in caregivers of other cultures. With an increasing immigrant population in the United States (Alperin & Batalova, 2018), more studies are needed to address how to best provide support for caregivers of PWD, given their unique migration experiences and histories, in order to better improve well-being for the community as a whole.

## Acknowledgments

The study was supported by Rupe Foundation (Principal Investigator: Jung-Ah Lee, PhD, RN) and University of California, Irvine Undergraduate Research Program (fellow – Christine Le). We thank Pauline Le, MSW, Family Caregiver Resource Center, Tricia Nguyen, CEO of Southland Integrative Services, Inc, and local physicians for their support in recruitment. We also thank bilingual students (Hoa Diep, Vianh Hoang, Diana Tran, Tiffany Tran) for their time and support for the home visits to Vietnamese family caregivers.

## Declaration of Conflicting Interests

The authors declared no potential conflicts of interest concerning the research, authorship, or publication of this article.

## Funding

This study was supported by Arthur N. Rupe Foundation and University of California Irvine Undergraduate Research Program.
